# Online eurythmy therapy for cancer-related fatigue: a prospective repeated-measures observational study exploring fatigue, stress, and mindfulness

**DOI:** 10.3389/fnint.2024.1472562

**Published:** 2024-09-19

**Authors:** Eliane Timm, Yobina Melanie Ko, Theodor Hundhammer, Ilana Berlowitz, Ursula Wolf

**Affiliations:** ^1^Institute of Complementary and Integrative Medicine, Faculty of Medicine, University of Bern, Bern, Switzerland; ^2^Eurythmy4you, Nidau, Switzerland

**Keywords:** fatigue, cancer, cancer-related fatigue, mindful movement, mindfulness-based, eurythmy therapy, integrative medicine, online intervention

## Abstract

**Introduction:**

Cancer is a debilitating disease with an often chronic course. One of the most taxing and prevalent sequelae in this context is cancer-related fatigue (CRF) resulting from the disease and/or associated treatments. Over the last years mindfulness-based interventions such as eurythmy therapy (ERYT), a mindful-movement therapy from anthroposophic medicine, have emerged as promising adjunct therapies in oncology. This prospective study investigated an online implementation of ERYT for CRF using a single arm repeated-measures design based on two consecutive studies.

**Method:**

Study 1 consisted of an initial assessment before, during, after, and at follow up of a 6-week online ERYT-based program in a mixed sample of *N* = 165 adults with or without cancer diagnosis. Study 2 involved a similar design with an adapted 8-week online ERYT-based program in a sample of *N* = 125 adults who had been diagnosed with cancer. Outcomes were assessed using the Functional Assessment of Chronic Illness Therapy–Fatigue, Perceived Stress Scale, Mindful Attention Awareness Scale, and Insomnia Severity Index (for Study 1 all, for Study 2 only the former three). We additionally performed an exploratory analysis regarding practice frequency and duration. Data were analyzed using Linear Mixed-Effect Models per outcome; ANOVA was used for practice times.

**Results:**

For Study 1, mixed-effects model estimates showed no significant effect on fatigue, but pointed to significantly improved emotional and physical well-being, reduced stress, as well as increased mindfulness (mixed subjects). Functional and social well-being or sleep quality did not change significantly. Study 2 model estimates on the other hand showed significantly improved CRF in conjunction with the ERYT-based online intervention, as well as improved stress and mindfulness scores (cancer-diagnosed subjects).

**Conclusion:**

Taken together, while our results should be interpreted with caution given the single-arm design and relatively high dropout, they suggest online ERYT may be associated with a reduction in fatigue for individuals diagnosed with cancer, an increase in mindfulness, and benefits for stress and certain well-being indicators. The online group format is advantageous in view of affordability and accessibility, the latter being particularly relevant for individuals who due to high symptom severity cannot leave their homes. Randomized-controlled studies will be needed to confirm these findings.

## 1 Introduction

Cancer is a complex and debilitating disease with an often chronic course, affecting millions of individuals worldwide (Tran et al., 2022; Sung et al., 2021). One of the most prevalent and taxing sequelae in this context is cancer-related fatigue (CRF), a general and persistent lack of energy not relieved by rest, which results from both the disease and associated treatments (Bower, 2014; Cleeland et al., 2013). Reported prevalence rates of CRF depend on type of treatment, population, and assessment methodology, but may range between 25 and 99% (Bower, 2014) or 14–100% (Ma et al., 2020), with the latter meta-analysis reporting a pooled prevalence of 52% based on 84 studies.

Mind–body therapies and particularly mindfulness-based movement practices such as Yoga, Tai Chi, Qi Gong, or Feldenkrais, which combine bodily movements (and often also breath) with focused attention, have emerged as promising adjunct therapies in oncology to improve common sequelae like CRF, sleep disturbances, reduced quality of life, or stress (Chen et al., 2024; Firkins et al., 2020; Mayden, 2012; Zhang et al., 2019; Wu et al., 2022; Desveaux et al., 2015; Raman et al., 2013; Gouw et al., 2019; Wang et al., 2013; Hillier and Worley, 2015; Cramer et al., 2012b; Vergeer et al., 2021; Miller et al., 2020; Stephens and Hillier, 2020; Wang and Szabo, 2020; Cocchiara et al., 2020). Eurythmy therapy (ERYT) is an integrative movement-based therapy in this context rooted in anthroposophic medicine, which involves integrated sequences of movements, performed with arms, hand, legs, or the whole body, in a state of focused concentration and intentionality (Kirchner-Bockholt, 1977; Kienle et al., 2013). Guided by a trained therapist, the technique is said to develop mindfulness to outer movement, inner sensation, and the connection between the two (Berger et al., 2015), a process engaging both proprioceptive and interoceptive awareness. Generally speaking, ERYT protocols can be practiced individually or in groups, and consist of movement sequences based on a core set of principles which are then tailored to a given disease or patient at hand (Hamre H. J. et al., 2007). Emerging scientific evidence points to benefits for a range of conditions (Lötzke et al., 2015) such as stress (Berger et al., 2015; Kanitz et al., 2011), depression (Hamre et al., 2006; Hamre et al., 2013), anxiety (Schwab et al., 2011; Hamre et al., 2009), or chronic pain (Hamre H. et al., 2007; Michalsen et al., 2021). Indeed, ERYT has also been applied in complementary cancer care, with promising first results (Kanitz et al., 2013). A challenge in this context however remains that disease- and treatment-related symptoms, including CRF, often restrict patients’ ability to travel to practice sessions (Kim et al., 2020). Online delivery of ERYT could hence be a promising avenue in oncology, as it allows patients to engage in the therapeutic activity without requiring them to leave their homes. Indeed, online applications of other mindful movement practices, such as Tai Chi or Qi Gong, have been shown to present useful alternatives in similar and other relevant contexts (Oh et al., 2021; Gao et al., 2022; Sohl et al., 2024; Brosnan et al., 2021; Gravesande et al., 2023; Teo et al., 2024). Nonetheless, research assessing the online delivery of ERYT in particular to our knowledge has not yet been conducted.

The current study thus assessed an online intervention based on ERYT to improve CRF and associated symptoms. More specifically, using an exploratory approach with repeated-measures design based on two consecutive observational studies, we aimed to assess changes in CRF (main outcome), sleep quality, stress, quality of life indicators, and mindfulness (secondary outcomes) during and after an online ERYT-based intervention for adults diagnosed with cancer. The study was conducted by the University of Bern (Institute of Complementary and Integrative Medicine) in collaboration with a healthcare provider specializing in ERYT.^1^

## 2 Study 1: exploratory assessment (mixed subjects)

### 2.1 Study 1 methods

#### 2.1.1 Study design and setting

The first study (11/2022–2/2023) involved an observational assessment with repeated-measures design on a mixed subjects sample, focusing on fatigue, sleep quality, mindfulness, quality of life indicators, and perceived stress as outcomes. The study included five measurement points, namely t1 at baseline, t2–t3 during the intervention, t4 just after completion of the intervention, and t5 at follow up. All surveys were conducted online and in full anonymity of participants. Anonymous self-generated codes were used to link an individuals’ repeated measures between assessment times. Given the design was observational and no potentially identifying data were collected (no names, email or IP addresses, birthdates, etc.), ethics approval was not required for this study according to the responsible Ethics Committee guidelines and the Federal Act on Research involving Human Beings (Human Research Act, 2011).

#### 2.1.2 Participants and procedure

Information about the intervention as well as the study were announced on the health provider’s website, newsletters, in psychology networks, adverts in clinics also practicing anthroposophic medicine, physicians’ practices, and social media. All individuals who registered for the two cycles of the intervention (one held in German and one in English) between November and December 2022 were invited to participate in the study. They were thoroughly informed about the study, specifying that participation was voluntary and that opting not to take part in the study would not impact their enrollment and participation in the intervention itself. In view of the exploratory aim of Study 1, we included all individuals who registered for the program and agreed to participate in the study. This meant that also individuals who did not have a cancer diagnosis but enrolled in the program for other reasons (e.g., relatives of cancer patients) and agreed to participate were included. Except for the intervention itself (free of charge), no compensation was offered to participants. All participants were asked to fill in the baseline survey at the outset of the intervention (t1), 2 weeks later (t2), 4 weeks later (t3), 6 weeks later (t4, which marked the completion of the intervention), as well as at follow-up (t5) after 14 weeks (i.e., 8 weeks after completion).

#### 2.1.3 Measures

The online survey was made of a set of validated psychological questionnaires programmed by means of SoSci Survey (Leiner, 2023) for anonymous computer-assisted implementation, and was available in German and English. For the validated scales the recall period was set to the last 7 days. The survey further included single items to indicate age, gender, cancer status, motive for enrolment, and practice times. For self-reported practice times we used the following quantitative items (t2–t5 assessment: *how many days in the last 2 weeks*/at follow-up: *per week on average*; *were you able to do the exercises?* answer options: *0–2 days*, *3–5 days*, *6–8 days*, *9–11 days*, *12–14 days*; and, respectively, *0–1 days*, *2–3 days*, *4–5 days*, and *6–7 days*). They were also asked how much time they have spent on the exercises per practice day (*1–10 min*, *11–20 min*, *21–30 min*, *more than 30 min*, or *not done*). *Functional Assessment of Chronic Illness Therapy - Fatigue:* To assess CRF and associated quality of life/well-being indicators we used the Functional Assessment of Chronic Illness Therapy–Fatigue (FACIT-F; Cella, 1997, validated German version: Montan et al., 2018; facit.org, 2022). The 40 items of this instrument were rated on a five-point Likert scale ranging from 0 (*not at all*) to 4 (*very much*). The overall FACIT-F score was calculated by summing the item scores (range 0–160), with higher scores indicating less fatigue/higher quality of life. The instrument includes five subscales, namely Fatigue (FA; 13 items), Physical Well-Being (PWB; 7 items), Social/Family Well-Being (SWB; 7 items), Emotional Well-Being (EWB; 6 items), and Functional Well-Being (FWB; 7 items). Most pertinently here, the FA subscale can range from 0 to 52, with lower scores implying more fatigue (clinical cut-off at 36; Alexander et al., 2009). *Mindful Attention Awareness Scale:* The Mindful Attention Awareness Scale (MAAS; Brown and Ryan, 2003; Carlson and Brown, 2005, validated German version: Michalak et al., 2008) is a widely used brief mindfulness questionnaire that showed good psychometric properties in cancer patient samples (Tseng, 2024). It is made of 15 items, each presenting a statement related to mindful awareness rated on a scale from 1 (*almost always*) to 6 (*almost never*). The MAAS score is the mean of all items and ranges from 1 to 6, with higher scores indicating higher mindfulness. *Perceived Stress Scale:* The Perceived Stress Scale (PSS-10; Cohen et al., 1983, validated German version: Klein et al., 2016; Copyright © 2022 Mapi Research Trust) is a well-established validated questionnaire for assessing perceived stress, which has also been extensively used in the context of cancer patients (Yılmaz Koğar and Koğar, 2024; Tseng, 2024; Chui, 2021; Chrobak et al., 2023; Trojnar et al., 2024; Soria-Reyes et al., 2023). Each of the 10 items is rated on a five-point Likert scale from 0 (*never*) to 4 (*very often*), with higher scores indicating greater perceived stress (total score calculated by summing the items, range: 0–40). PSS-10 total scores can be interpreted as low (0–13), moderate (14–26), or high (27–40) stress (Adamson et al., 2020). *Insomnia Severity Index*. The Insomnia Severity Index (ISI; Morin et al., 2011; Morin, 1993, German version: Dieck et al., 2018; Copyright © 2022 Mapi Research Trust) is a 7 item instrument assessing the perceived severity of insomnia. It has been frequently used in research on cancer populations with good validity and reliability (Yusufov et al., 2019; Lin et al., 2020; Michaud et al., 2021; Savard et al., 2005). Each item is rated on a five-point Likert scale from 0 to 4. The total score (calculated by summing the items) ranges from 0 to 28, with higher scores indicating greater severity (i.e., worse sleep quality).

#### 2.1.4 Intervention

The mindful movement-based intervention consisted of weekly online group sessions (90 min) and guided self-practice between sessions over six consecutive weeks. In the online sessions participants were familiarized with a specific sequence of ERYT exercises tailored to address cancer-related symptoms and CRF (for further information on ERYT in the context of cancer and CRF, see Kröz et al., 2013; Meier-Girard et al., 2020; Kröz et al., 2017), conveyed step by step over the course of the program. In a given session, the facilitator (an experienced and certified eurythmy therapist; TH) demonstrated each of the exercises at full length live on camera, while also verbally describing the specifics of the movements and pointing out relevant meaning content. After some initial rounds of demonstration, participants were encouraged to actively join the facilitator, gradually imitating the movements until they were able to perform the exercise independently. This practice time (30–40 min) was followed by a short resting period (5 min), during which the facilitator guided participants through a body scan, aimed to enable them to sense directly in their body the impact of the exercise. Participants then had the opportunity to ask questions. The facilitator finally provided instructions on how to perform the exercise on their own in their forthcoming self-practice during the time lapse between sessions, including what to pay specific attention to, context of practice, and duration, with a recommendation of 10–15 min of daily self-practice. In the subsequent session, the facilitator encouraged participants to discuss their experiences and ask questions if needed. Furthermore, participants had access to video recordings of the live sessions and of demonstrations of specific ERYT exercises, as well as to an online group forum for exchange.

#### 2.1.5 Data analysis

All statistical analyses were performed using *R* version 4.4.0 (R Core Team, 2024). For all inferential statistics the significance level was set to *α* < 0.05. Surveys that had been filled in outside the defined time windows (for t1 more than 1 week after intervention onset, for t2–t4 less than one or more than 3 weeks apart, and for t5 outside the required period of 4–10 weeks post intervention end) where excluded from the analysis. To analyze time-dependent changes in the outcome variables and test for significance we performed Linear Mixed-Effects Models (LMM) (Brown, 2021) using the *R* packages *lme4* (Bates et al., 2015) and *nlme* (Pinheiro et al., 2023). We opted for LMM among other reasons due to its capacity to calculate accurate models in spite of missing data, a common challenge in longitudinal studies (Gabrio et al., 2022). Models were calculated for each outcome separately, with all models adjusted for age, gender, and survey language. Finally, to test the effect of self-practice time on the outcomes we performed explorative one-way Analysis of Variance (ANOVA) with Tukey post-hoc tests for practice frequencies and duration for each measurement point, starting with t2.

### 2.2 Study 1 results

#### 2.2.1 Subjects

**Figure 1:**
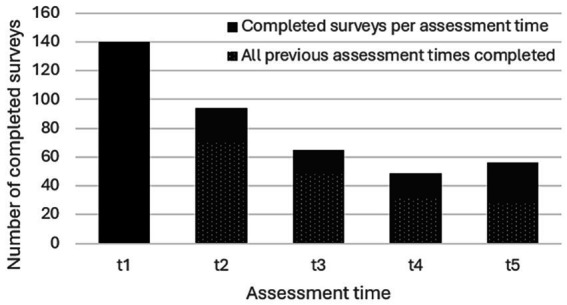
Number of completed surveys per assessment time (Study 1).

Of the 283 individuals who registered for the program in Study 1 (187 for the English and 96 for the German iteration), *N* = 165 agreed to participate in the study and filled in at least one questionnaire (57.6% filled in at least two questionnaires and 17% filled in all five questionnaires, see Figure 1). About two thirds of participants (*n* = 99) filled in the English version of the survey, the rest used the German version (*n* = 66). Sociodemographic characteristics of Study 1 participants are given in Table 1. The mean age was 59 years (*SD* = 12.2), a large majority being female. Nearly a third (*n* = 52) reported to have received a cancer diagnosis during their lifetime, of which 26 participants indicated to be currently in treatment. Means and standard deviations of Study 1 outcome variables at each of the five assessment times can be found in Table 2. The subjects’ baseline FACIT-F FA score was above 36 and can thus be considered non-clinical (Alexander et al., 2009). Baseline PSS-10 scores pointed to moderate stress levels (Adamson et al., 2020), and baseline MAAS scores can be considered within the normative range based on a large-scale norming study (Brown and Kasser, 2005; Carlson and Brown, 2005). Finally, the subjects’ baseline ISI scores suggest mild to moderate severity of insomnia as per clinical benchmarks (Morin et al., 2011).

**Table 1:** Age and gender of subjects (Study 1).

	*n*
Age groups (in years):	
21–40	11
41–50	32
51–60	39
61–70	56
71–97	25
n.a.	2
Mean sample age (*SD*)	59.0 (12.24)
Gender	
Female	127
Male	17
Diverse	0
n.a.	21

n.a., response not available.

**Table 2:** Means and standard deviations of all outcome variables per measurement point (Study 1).

	t1		t2		t3		t4		t5	
	*n*	*M* (*SD*)	*n*	*M* (*SD*)	*n*	*M* (*SD*)	*n*	*M* (*SD*)	*n*	*M* (*SD*)
FACIT-F total	128	116.41 (24.92)	62	117.8 (22.93)	47	117.11 (23.42)	35	117.57 (25.47)	41	119.1 (25.12)
–PWB	133	22.76 (5.32)	84	23.86 (4.13)	61	23.86 (4.52)	48	23.14 (5.25)	52	23.35 (4.39)
–SWB	128	18.50 (6.18)	74	17.36 (6.58)	60	19.03 (6.15)	45	18.44 (7.02)	49	19.10 (5.63)
–EWB	132	17.46 (5.05)	65	18.61 (3.94)	49	18.64 (3.58)	37	18.64 (4.20)	47	19.11 (3.84)
–FWB	132	19.59 (6.18)	80	20.04 (4.98)	62	19.92 (5.23)	47	20.23 (4.84)	54	20.35 (5.35)
–Fatigue	132	37.95 (10.20)	80	39.72 (8.89)	62	39.11 (9.58)	47	40.27 (9.41)	53	38.93 (9.72)
PSS-10	130	15.42 (6.87)	80	13.81 (5.82)	63	13.65 (6.74)	46	13.00 (6.08)	52	13.37 (6.11)
MAAS	128	4.22 (0.86)	79	4.40 (0.81)	63	4.53 (0.79)	48	4.81 (0.78)	54	4.67 (0.71)
ISI	128	8.36 (5.85)	81	7.45 (5.74)	62	8.27 (5.62)	48	7.27 (5.72)	54	8.13 (6.47)

FACIT-F, Functional Assessment of Chronic Illness Therapy–Fatigue (facit.org, 2022), total score range: 0–160; PWB, Physical Well-Being (range 0–28); SWB, Social Well-Being (range 0–28); EWB, Emotional Well-Being (range 0–24); FWB, Functional Well-Being (range 0–28), Fatigue (range 0–52); PSS-10, Perceived Stress Scale (Cohen et al., 1983), range 0–40; MAAS, Mindful Attention Awareness Scale (Brown and Ryan, 2003; Carlson and Brown, 2005), range 1–6; ISI, Insomnia Severity Index (Morin, 1993; Morin et al., 2011), range 0–28. t1 = baseline, t2/t3 = during, t4 = at completion, t5 = follow-up.

#### 2.2.2 Time-dependent changes in outcome variables

**Figure 2:**
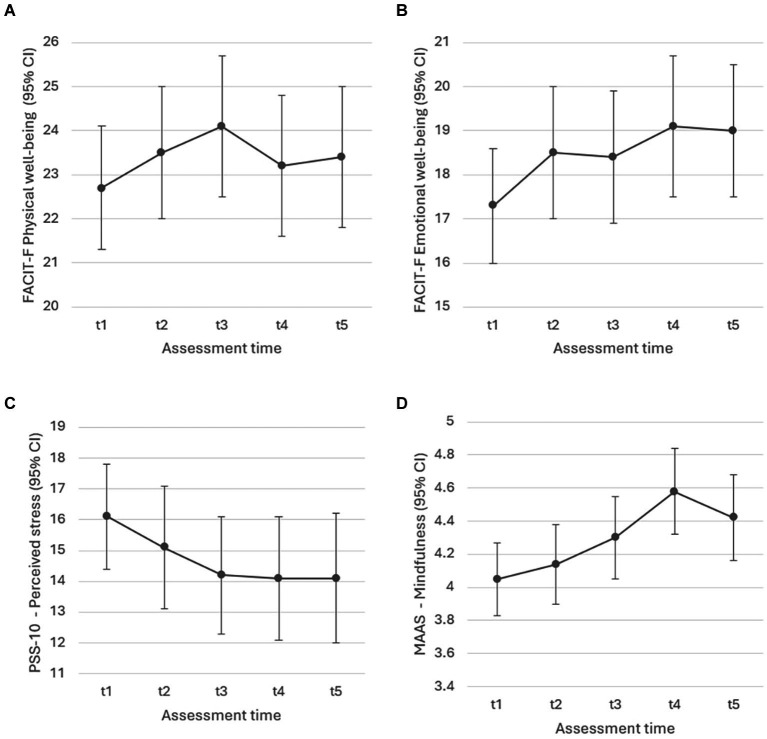
Study 1: Liner Mixed-Effect Model estimates with **(A)** physical well-being (*p* = 0.029), **(B)** emotional well-being (*p* < 0.001), **(C)** perceived stress (*p* = 0.003), and **(D)** mindfulness (*p* < 0.001) as outcomes.

Figure 2 shows significant changes in the outcomes over time found in Study 1. *CRF and quality of life indicators.* There was a significant improvement in the FACIT-F physical well-being (*F*(4, 197) = 2.764, *p* = 0.029) and emotional well-being (*F*(4, 158) = 5.181, *p* < 0.001) scales (Figures 2A,B, respectively). The FACIT-F fatigue subscale score also improved, but not significantly (*F*(4, 198) = 1.948, *p* = 0.104). The same was the case for social (*F*(4, 180) = 1.831, *p* = 0.125) and functional (*F*(4, 199) = 1.227, *p* = 0.301) well-being and the overall FACIT-F score (*F*(4, 144) = 2.362, *p* = 0.056). For EWB all estimates were significant relative to t1 at *p* < 0.001, except for t2 (*p* < 0.01) and t3 (*p* < 0.05). For PWB only t2 (*p* < 0.05) and t3 (*p* < 0.01) estimates were significant relative to t1. *Perceived stress*. Figure 2C shows changes in the PSS-10 scores, pointing to a significant reduction in stress over the course of the intervention (*F*(4, 198) = 4.110, *p* = 0.003). Except for t2 (n.s), all estimates were significant relative to t1 at *p* < 0.01. *Mindfulness.* Time-dependent changes in mindfulness are visible in Figure 2D, with MAAS scores increasing significantly over the course of the intervention (*F*(4, 200) = 12.467, *p* < 0.001). The estimates were significant relative to t1 at *p* < 0.001, except for t3 (*p* < 0.01) and t2 (n.s.). *Sleep*. Sleep quality as per ISI did not significantly change in conjunction with the intervention (*F*(4, 201) = 1.724 *p* = 0.146).

#### 2.2.3 Effects of self-practice frequency and duration

In Study 1, the frequency of practice (number of days practiced during the past 2 weeks/during 1 week on average in case of t5) had a significant effect on stress levels as per ANOVA at t4 (*F*(4, 41), *p* = 0.031), with post-hoc tests showing significantly lower stress if 12–14 rather than 6–8 days had been practiced (*p* = 0.021). The duration of self-practice (minutes per day) in the preceding weeks also had a significant effect on stress at t4 (*F*(4, 41) = 3.038, *p* = 0.028), but only a trend of higher stress if over 30 min per day were practiced compared to 11–20 min (*p* = 0.073). As with stress, practice frequency of preceding weeks also had a significant effect on SWB at t4 (*F*(4, 40) = 2.738, *p* = 0.042). Post-hoc tests showed significantly higher SWB if 9–11 rather than 6–8 days had been practiced (*p* = 0.041). Furthermore, the duration of self-practice had a significant effect on SWB at t2 (*F*(4, 65) = 3.316, *p* = 0.016), with significantly higher SWB if they had practiced 1–10 min per day (*p* = 0.009) or 11–20 min per day (*p* = 0.035) during preceding weeks rather than no practice at all. Similarly, practice duration had a significant effect on FWB at t4 (*F*(4, 42), *p* = 0.021), with significantly lower FWB if they had practiced more than 30 min compared to 11–20 min during preceding weeks (*p* = 0.022). Finally, self-practice duration had a significant effect on sleep quality at t2 (*F*(4, 71), *p* = 0.034), with significantly better sleep quality if they had practiced 11–20 min compared to no practice at all (*p* = 0.033), and a significant effect at t4 (*F*(4, 43), *p* = 0.010) in which sleep quality was worse if they had practiced more than 30 min compared to 11–20 min during preceding weeks (*p* = 0.009).

### 2.3 Study 1 discussion

Study 1 assessed outcomes of a 6-week online intervention employing ERYT for CRF. While we found significant improvements in emotional and physical well-being, perceived stress, as well as mindfulness, there were no significant changes in fatigue, sleep quality, or social and functional well-being. The lack of significant changes in fatigue could be related to the subjects’ non-clinical degree of baseline fatigue. The sample’s baseline scores pointed to slightly more fatigue compared to normative values from healthy adults (Webster et al., 2003) but slightly less fatigue (although only by one point) than cancer-specific reference scores from a large-scale population survey (Butt et al., 2010). Indeed, the rather small proportion of cancer patients/survivors in the sample does not allow conclusions regarding CRF *per se*, a limitation of Study 1 which we subsequently addressed in Study 2. A further limitation of the study was the relatively high dropout between assessment times, which is common in online survey-based research, but could potentially give rise to bias and hence advises caution for the interpretation of the data. As with fatigue, baseline PSS-10 scores were somewhat above general population norms (Cohen, 1988) but below reference scores based on a breast cancer sample (Soria-Reyes et al., 2023). ISI scores were in contrast largely in line with cancer-specific normative values (Savard et al., 2005). Finally, the frequency and duration of self-practice of ERYT exercises beyond the practice during the guided online sessions appeared to play a role in shaping the magnitude of beneficial effects. While in several cases more frequent or longer self-practice times seemed to exert a favorable impact on the outcomes, this did not hold for all intervals, assessment times, or outcomes. Moreover, practice durations larger than 30 min in some cases even had a detrimental impact on sleep quality and functional well-being in the assessment thereafter, bearing in mind however that the exploratory design and modest number of cancer-diagnosed participants do not allow definitive conclusions with reference to causality and in general. Further research will be needed to establish optimal practice frequencies and durations per intervention phase. Taken together, the results suggest the online ERYT-based CRF intervention to merit further investigation; the findings from the first study were taken into account in the design of Study 2.

## 3 Study 2: assessment of CRF intervention (cancer-diagnosed subjects)

### 3.1 Study 2 methods

#### 3.1.1 Study design and setting

Study 2 (9/2023–2/2024) employed a similar design as the first study but focused on a sample diagnosed with cancer, with the assessment schedule and intervention adapted to the clinical requirements of the subjects at hand: In order to reduce participant burden we shortened the survey by limiting the FACIT-F to the fatigue subscale and dropping the ISI. Furthermore, by omitting one of the two assessment times during the intervention we reduced the number of surveys to be completed from 5 to 4. As in Study 1, all survey data was collected online and in full anonymity of participants (including anonymous self-generated codes to link the repeated measures) without collecting any potentially identifying information; the study hence did not require ethics approval. The main outcome of Study 2 was CRF, with stress and mindfulness as secondary outcomes.

#### 3.1.2 Participants and procedure

The study was announced in the same fashion as in Study 1. All individuals who registered to one of two cycles of the intervention (one held in German, one in English, and the latter also being simultaneously translated to Chinese and Indonesian with the support of two professional interpreters) between September and November 2023 were invited to participate in the study. As in Study 1, no compensation was offered to participants except for the free of charge intervention. They were thoroughly informed about the study, specifying that participation was voluntary and that opting out would not impact their participation in the intervention itself. Only those who indicated that they had been diagnosed with cancer within the last 5 years were included in the study. The choice of a 5-year period was based on evidence that CRF may persist for up to 5 years after treatment, or longer (Bower, 2014). All participants were asked to fill in the baseline survey at the outset of the intervention (t1), 4 weeks later (t2), 8 weeks later (t3–completion of the intervention), and 16 weeks later (i.e., 8 weeks after the end of the intervention) as a follow-up assessment (t4).

#### 3.1.3 Measures

We used the same measures as in Study 1 to assess CRF, stress, and mindfulness, using the original recall periods for all instruments. In addition to the English and German language versions, a Chinese version of the survey was prepared using validated translations of the FACIT-F fatigue subscale (facit.org, 2022; Cai et al., 2023), the PSS-10 (Jiang et al., 2023), and the MAAS (Chen et al., 2012). Finally, we again asked about frequency and duration of self-practice (e.g., *On average, how many days per week were you able to do the exercises*?).

#### 3.1.4 Intervention

Like in Study 1, the intervention consisted of weekly online group sessions, but the overall duration was extended to 8 weeks. Furthermore, the specific ERYT exercises shown to the participants were adapted to target fatigue more specifically and match an 8-weeks program, and additional behavioral exercises designed to foster mindfulness and self-awareness based on anthroposophic medicine (Haas, 2017) were incorporated into the intervention. As in Study 1, participants were encouraged to practice the learned exercises by themselves during the subsequent week, with a recommendation to practice at least 15 min daily, and were invited to use the video-recorded demonstrations of the exercises and a group forum.

#### 3.1.5 Data analysis

Except for the outcome variables that were dropped, the data analysis protocol was identical to the one described in Study 1. The defined time windows for filling in Study 2 surveys were adapted to match the number of measurement points; namely, surveys were excluded from the analysis if t1 survey was filled in more than 1 week after intervention start, t1–t3 surveys less than 3 or more than 6 weeks apart from each other, or t4 survey outside the required 4–10 weeks after t3 survey.

### 3.2 Study 2 results

#### 3.2.1 Subjects

**Figure 3:**
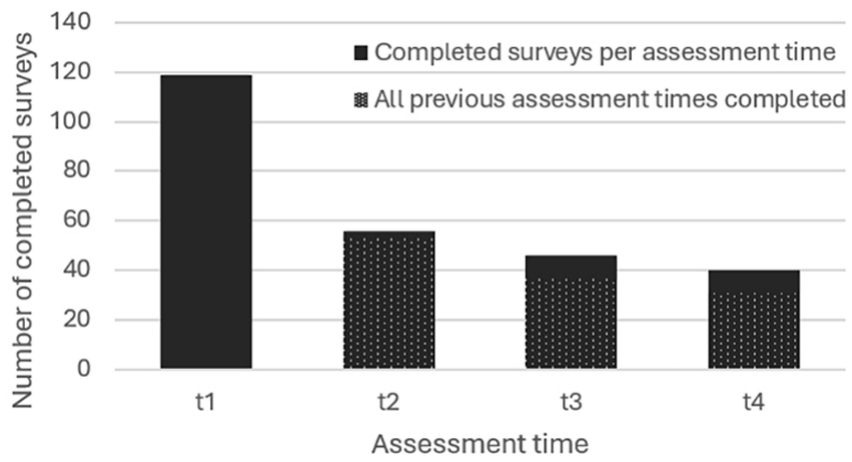
Number of completed surveys per assessment time (Study 2).

Of the 303 individuals who registered for the intervention in Study 2 (242 for the English, 61 for the German iteration), *N* = 125 agreed to participate in the study and filled in at least one survey. Of these subjects, 48% filled in at least two surveys and 24.8% filled in all four surveys (see Figure 3). About half of them used the English version of the survey (*n* = 64), slightly more than a third used the German (*n* = 46), and the remainder the Chinese version (*n* = 15). Table 3 shows sociodemographic characteristics of Study 2 participants. As in the former study, a large majority of participants were female, and the mean age was 56.57 years (*SD* = 11.87). Means and standard deviations of Study 2 outcome variables at all assessment times are found in Table 4. The subjects’ baseline FACIT-F fatigue subscale scores were below the cut-off of 36 (Alexander et al., 2009) which suggests a clinically significant degree of fatigue; they were also below reference values from a large cancer patients sample (Butt et al., 2010) and norms for healthy adults (Webster et al., 2003). Baseline PSS-10 scores were indicative of moderate stress levels (Adamson et al., 2020) comparable to reference scores of a large-scale cancer patients sample (Soria-Reyes et al., 2023) and above general population norms (Cohen, 1988). Finally, baseline MAAS scores of participants were somewhat below general population normative values (Brown and Kasser, 2005), as well as a large-scale sample of cancer patients (Carlson and Brown, 2005).

**Table 3:** Age and gender of the sample (Study 2).

	*n*
Age groups (in years)	
23–40	9
41–50	34
51–60	36
61–70	28
71–82	18
Mean sample age (*SD*)	56.57 (11.87)
Gender:	
Female	105
Male	16
Diverse	0
n.a.	4

n.a., response not available.

**Table 4:** Means and standard deviations for all outcome variables (Study 2).

	t1		t2		t3		t4	
	*n*	*M* (*SD*)	*n*	*M* (*SD*)	*n*	*M* (*SD*)	*n*	*M* (*SD*)
FACIT-F Fatigue	118	27.49 (11.11)	52	33.60 (9.61)	44	33.91 (10.27)	32	35.28 (10.09)
PSS-10	118	20.47 (6.63)	52	16.54 (6.08)	46	16.22 (6.20)	40	15.85 (6.67)
MAAS	117	3.84 (0.90)	51	4.19 (0.80)	45	4.27 (0.81)	40	4.47 (0.89)

FACIT-F, Functional Assessment of Chronic Illness Therapy–Fatigue subscale (facit.org, 2022); PSS-10, Perceived Stress Scale (Cohen et al., 1983), range 0–40; MAAS, Mindful Attention Awareness Scale (Brown and Ryan, 2003; Carlson and Brown, 2005), range 1–6; t1 = baseline, t2 = mid-term, t3 = at completion, t4 = follow-up.

#### 3.2.2 Time-dependent changes in outcome variables

**Figure 4:**
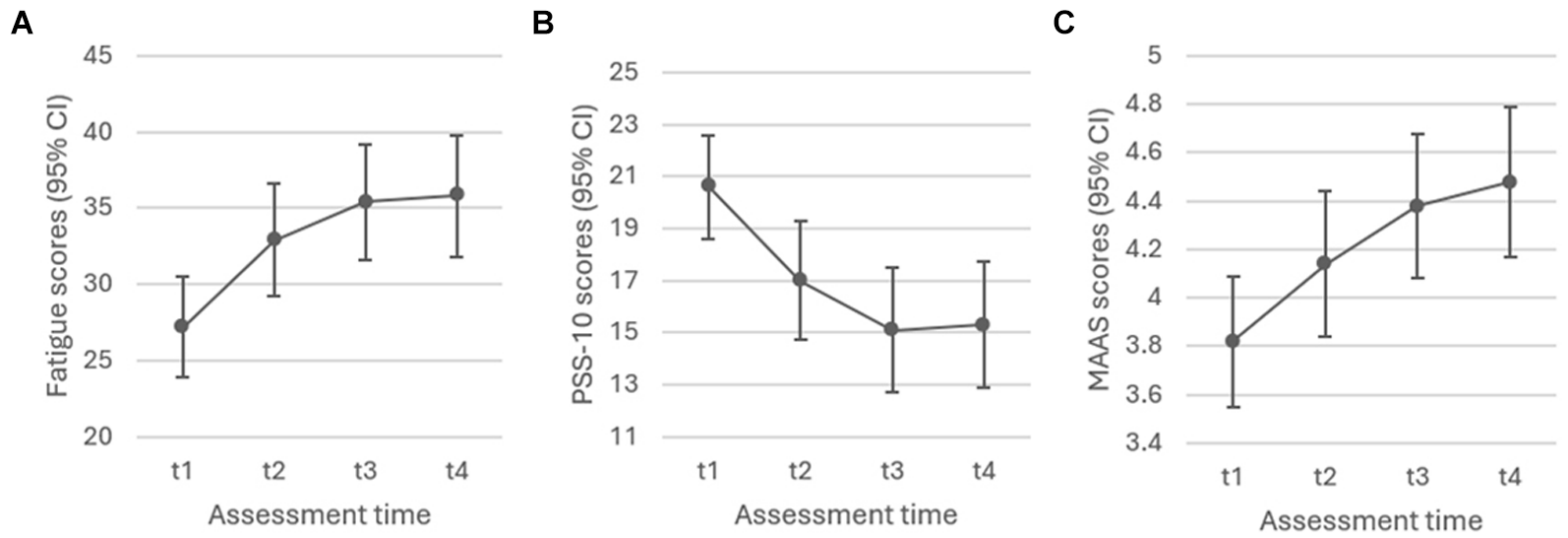
Study 2: Liner Mixed-Effect Model estimates with **(A)** fatigue (*p* < 0.001), **(B)** perceived stress (*p* < 0.001), and **(C)** mindfulness (*p* < 0.001) as outcomes.

*Cancer-related fatigue.* Fatigue as assessed by the FACIT-F fatigue subscale showed a significant decrease over the course of the intervention (*F*(3, 119) = 23.618, *p* < 0.001) in Study 2, as can be seen in Figure 4A. *Perceived stress*. Similarly, scores on the PSS-10 showed a significant reduction in stress over time (*F*(3, 129) = 22.414, *p* < 0.001), as depicted in Figure 4B. *Mindfulness*. Finally, Figure 4C shows MAAS scores per assessment time, pointing to a significant increase in mindfulness over the course of the intervention (*F*(3, 128) = 24.323, *p* < 0.001). For all three Study 2 outcomes, estimates (t2, t3, t4) were significant relative to t1 at *p* < 0.001.

#### 3.2.3 Effects of self-practice frequency and duration

As per ANOVA, the mean number of days per week of self-practice over the past 4 weeks/8 weeks in case of t4 (frequency) had a significant effect on stress in Study 2 at t3 (*F*(3, 42) = 4.248, *p* = 0.010), with 4–5 days of practice in the preceding weeks associated with less stress compared to 0–1 days (*p* = 0.006). Conversely, at t4 (*F*(3, 36) = 2.925, *p* = 0.047) 4–5 days of self-practice during the preceding weeks was associated with higher stress compared to 2–3 days of self-practice (*p* = 0.037). Self-practice frequency had a significant effect on mindfulness at t3 (*F*(3, 41) = 3.951, *p* = 0.015), with 4–5 days of self-practice associated with higher mindfulness scores than precedent self-practice of only 2–3 days (*p* = 0.045). Finally, the mean duration of self-practice per day in the preceding weeks had a significant effect on fatigue at t2 (*F*(4, 45), *p* = 0.037), with significantly more fatigue in conjunction with a practice of longer than 30 min compared to no practice at all (*p* = 0.024).

### 3.3 Study 2 discussion

Using a repeated-measures design with four assessment times (follow-up at 8 weeks post intervention) in subjects diagnosed with cancer (past 5 years), Study 2 found significantly improved CRF, stress, and mindfulness scores in conjunction with the adapted ERYT-based online intervention. The exploratory analysis of practice frequency and duration yielded inconclusive results. As with the former study, the interpretability of our findings is challenged by relatively high dropout rates, a common problem of online survey-based research. A detailed discussion of findings follows in the subsequent section.

## 4 General discussion

Persistent fatigue is one of the most common and taxing sequelae of cancer (Bower, 2014) and presents a challenge in a series of other chronic illnesses (Whitehead, 2009). Using an exploratory single-arm repeated measures design based on two consecutive studies, the current work assessed an online application of ERYT to address CRF and associated symptoms. Study 1 consisted of a preliminary assessment in a mixed subjects sample of *N* = 165 adults with or without cancer diagnosis. Mixed-effects model estimates of the repeated measures before, during, after, and at follow up of the 6-week ERYT online program pointed to significantly improved emotional and physical well-being, reduced stress, and increased mindfulness, but had no effect on fatigue, functional and social well-being, or sleep quality. However, given the low proportion of cancer patients and survivors in this first sample, conclusions regarding CRF could not be drawn based on Study 1. Study 2 therefore involved a similar design with a sample of *N* = 125 adults who had been diagnosed with cancer within the past 5 years. Model estimates showed significant improvements of CRF, stress, and mindfulness scores in conjunction with the adapted 8-week long ERYT-based online intervention.

To our knowledge this is the first study examining an online application of ERYT. Taken together, our results suggest that online ERYT may reduce CRF in individuals diagnosed with cancer, may involve an increase in mindfulness, and could be associated with benefits for stress and well-being. However, controlled clinical trials will be needed to confirm and further elaborate these findings. Clinical research on ERYT in general is still scarce, but our results are in line with the few existent face-to-face ERYT studies that found reduced CRF in breast cancer patients and survivors (Oei et al., 2021; Kröz et al., 2023). Another study found reduced fatigue scores in moderately stressed adults after receiving ERYT (Kanitz et al., 2012), and a systematic review on the effectiveness of ERYT in various clinical populations (e.g., cancer, hypertension, chronic low back pain, anxiety, and other indications) concluded the implementation of ERYT as an adjunct therapy to be associated with improvements in health (Lötzke et al., 2015). Furthermore, our results are in line with findings on other mindful-movement based practices: Meta-analyses and reviews on Yoga, Tai Chi, and Qigong for instance concluded that these practices were able to relieve CRF in cancer patients/survivors (Dong et al., 2019; Sadja and Mills, 2013; Cramer et al., 2012a; Armer and Lutgendorf, 2019; Liu et al., 2020; Song et al., 2018; Yin et al., 2020; Zeng et al., 2019), but described magnitude of effects varied from study to study. Furthermore, our results converge with meta-analytic findings reporting significantly reduced CRF in RCTs of Mindfulness-Based Stress Reduction (MBSR, Kabat-Zinn, 2003) and similar interventions in oncology populations (Johns et al., 2021; Chayadi et al., 2022; Xie et al., 2020; Zhang et al., 2019; McCloy et al., 2022), although one meta-analysis found improved stress and sleep but no significant effects on fatigue or quality of life in conjunction with MBSR (Wu et al., 2022). In several of the former meta-analyses results regarding sleep quality were less consistent (Zhang et al., 2019; McCloy et al., 2022), which was perhaps mirrored in our Study 1, although, as mentioned before, the sample did not allow conclusions regarding oncology populations. Finally, we found a series of significant but inconclusive effects of differential practice times (frequencies and durations) of ERYT on CRF, stress, and mindfulness (Study 2), as well as sleep and well-being indicators (Study 1), but only for selected assessment times, and the optimal amount of self-practice appeared to differ in relation to the various assessment times and outcome variables. While further research with systematic comparisons of self-practice times will be needed to elucidate these findings, the current exploratory assessment suggests a tendency of more/longer self-practice associated with benefits, although in a few instances the opposite was the case. Given the latter occurred particularly in the context of extended self-practice times, it is not inconceivable that lengthening practice beyond a certain optimum could turn benefit into detriment. Several reviews of mindfulness-based interventions report positive associations between home practice and intervention outcomes in both general and cancer-affected populations (Parsons et al., 2017; Baydoun et al., 2021; Kang et al., 2021), but research in this context is scarce, and a consensus on optimal practice times in view of treatment effectiveness and adherence remains to be established.

Although single-arm evaluations are an important step in exploratory research, the lack of a control condition and randomization was a limitation of the current work and will be necessary to confirm beneficial effects of online ERYT. Furthermore, due to the voluntary nature of participation and hence self-selection of participants we cannot rule out sampling bias, which limits the generalizability of our findings. Finally, response and survey completion rates per assessment time (Figures 1, 3) were relatively low, which is however not unusual for online studies based on voluntary and non-compensated participation (Rostaminezhad et al., 2013; Bawa, 2016; Fish et al., 2016; Meyerowitz-Katz et al., 2020), but nonetheless speaks for cautious interpretation of results. Indeed, the possibility of dropout effects represents a notorious challenge for clinical research relying on longitudinal assessments; such effects may compromise interpretability of findings in particular, in cases in which the dropout-related missingness is non-random (Bell et al., 2013), which is generally difficult to rule out, but for which mixed-effect models are considered a method of choice, given their capacity to minimize dropout bias in longitudinal research with both random or non-random missingness (Mallinckrodt et al., 2001; Bell et al., 2013). Although our findings provide promising first indications, future studies should hence attempt to reduce dropout (Meyerowitz-Katz et al., 2020), include controlled designs, as well as systematic comparisons of varying practice times and subgroups (e.g., individuals currently in treatment vs. cancer survivors). In addition, assessing the duration of disease and/or remission period as well as therapeutic protocols per participant would be favourable for an enhanced contextualization of CRF levels and other outcomes at the various assessment times. Strengths of the current work include the novelty of the approach and its potential to expand the still limited toolkit for addressing CRF in oncology populations, as well as the limited research on ERYT in general. Furthermore, the group format and online delivery imply affordability and scalability, which along with the adaptability of ERYT exercises, renders the intervention more broadly accessible, also in locations in which ERYT therapists are not readily available, and, pertinently, for individuals who are unable to leave their home due to debilitating cancer treatment side-effects (e.g., dizziness, nausea, swollen joints; Kim et al., 2020) and not least CRF itself (Bower, 2014). A recent meta-analysis of online interventions involving mindfulness-based practices for cancer patients concluded online delivery to be both feasible and acceptable to manage cancer-related symptoms (Fan et al., 2023), with similar conclusions reiterated in studies examining online delivery of specific mindfulness-based movement practices, such as Tai Chi and Qigong (Oh et al., 2021; Gao et al., 2022; Sohl et al., 2024). Online ERYT may be an additional such option, with promising first results that should be further investigated.

## Data Availability

The raw data supporting the conclusions of this article will be made available by the authors, without undue reservation.

## Footnotes

^1^ *Eurythmy4you.*
